# Reduction in the utilization of prednisone or methotrexate in Canadian claims data following initiation of etanercept in pediatric patients with juvenile idiopathic arthritis

**DOI:** 10.1186/s12969-019-0358-x

**Published:** 2019-09-10

**Authors:** Majed Khraishi, Brad Millson, John Woolcott, Heather Jones, Lisa Marshall, Nicolino Ruperto

**Affiliations:** 1 Memorial University of Newfoundland, St. Johns, NL Canada; 2 IQVIA, Kanata, ON Canada; 3 Global Outcomes and Evidence, Pfizer, Collegeville, PA USA; 4 Global Medical Affairs, Pfizer, Collegeville, PA USA; 5 IRCCS, Istituto Giannina Gaslini, Clinica Pediatrica e Reumatologia – PRINTO, Genoa, Italy

**Keywords:** Juvenile idiopathic arthritis, Etanercept, Methotrexate, Prednisone, Claims data, Canada

## Abstract

**Background:**

In adult patients with arthritis, use of the tumor necrosis factor (TNF) inhibitor etanercept (ETN) is often associated with a reduction in the utilization of co-medications, particularly steroids. Comparatively little is known about the utilization of co-medications when ETN is initiated in pediatric patients with juvenile idiopathic arthritis (JIA).

**Methods:**

This study analyzed Canadian longitudinal claims level data spanning January 2007 to April 2017. Data were collated from the IQVIA Private Drug Plan, Ontario Public Drug Plan, and the Quebec Public Drug Plan (Régie de l’assurance maladie du Québec) databases. Patients < 18 years of age were indexed when filling a prescription for ETN between January 2008 and January 2016. Those who met the inclusion and exclusion criteria were assessed for methotrexate (MTX), and prednisone (PRD) use in the 6 months prior to and 12 months following initiation of ETN.

**Results:**

Longitudinal claims data for 330 biologic-naive pediatric patients initiating ETN therapy were included. The majority of patients were female (67%), aged 10–17 years (64%), and with a drug history consistent with JIA (96%). Most patients were from Quebec (36%) or Ontario (33%). Dosing of ETN was weight-based with a mean dosage over the first year of 31 mg per week. ETN dosing was relatively consistent over the first year. In total, 222 (67%) patients did not use MTX and 223 (68%) did not use PRD before or after starting ETN. A total of 17% (18/103) of MTX-treated and 50% (46/92) of PRD-treated patients discontinued use of those medications upon initiation of ETN treatment. In patients continuing MTX or PRD, significant reductions in the weekly dosage from 14.3 to 6.8 mg per week for MTX and from 56 to 23 mg per week for PRD were observed (*P* < 0.01).

**Conclusions:**

This study of Canadian claims-level data is the first large prespecified analysis of co-medication utilization following the initiation of ETN therapy in pediatric patients. A decline in both MTX and PRD use and dosage was observed and may be associated with benefits related to safety, tolerability, and overall healthcare costs.

## Background

Tumor necrosis factor alpha (TNFα) is a pro-inflammatory cytokine with a strong role in the pathogenesis of inflammatory diseases. [1] Biologic inhibitors of TNFα have shown significant efficacy in the treatment of several inflammatory diseases and they are now routinely used to manage rheumatic conditions, including juvenile idiopathic arthritis (JIA).

Patients with several types of JIA have shown significant reductions in both disease symptoms [2] and radiographic progression [3] when treated with etanercept (ETN), a TNF inhibitor. ETN is often used as a first-line biologic therapy for JIA [4, 5] and has been associated with disease quiescence in up to ~ 50% of ETN-treated children [5].

In Canada, ETN is approved for the treatment of patients aged 4–17 years with moderate to severe JIA and an inadequate response to ≥1 disease-modifying anti-rheumatic drugs (DMARDs) [6]. ETN is also approved for the treatment of chronic severe plaque psoriasis in patients aged 4–17 years who are candidates for systemic therapy or phototherapy, and pediatric patients with active ankylosing spondylitis [6]. Previous research in the Canadian setting has shown that annual retention of ETN treatment among pediatric patients with a medical history consistent with JIA (94%) or ankylosing spondylitis (5%) ranges from 78% in year 1 to 80 to 90% over years 2–6, [7] suggesting therapeutic efficacy and tolerability. These levels of ETN treatment retention are higher than those reported by adult patients in the same setting over the same time periods [7, 8].

In adults, it is recognized that biological therapy for inflammatory arthritis is often associated with a reduction in the requirements for other medications, most notably steroids, [9–14] which can reduce the incidence of side effects [15]. In children, long-term steroid use has been associated with a range of adverse events, including growth retardation and bone demineralization, [16–19] which can also result in a significant increase in healthcare utilization and cost [20–22]. The importance of avoiding long-term use of steroids has been highlighted in recent recommendations of an international JIA task force [23]. Little is known about the effect of ETN initiation on the utilization of steroids and other co-medications in pediatric patients with JIA or related conditions. According to a small survey (*n* = 82) of US pediatric physicians, a ≥50% reduction in disease activity was observed in 46% of patients with refractory systemic onset juvenile rheumatoid arthritis during the first 25 months of ETN therapy, with 46% of patients being able to discontinue steroid therapy [24]. However, it is not clear if similar benefits occur in pediatric patients with JIA in other clinical settings and regions.

The aim of this study was to use Canadian claims-level data to evaluate the utilization of the DMARD methotrexate (MTX) and PRD in Canadian pediatric patients with JIA who initiated ETN therapy.

## Methods

### Data sources

In Canada, while the majority of health services are paid through the provincial health ministries, prescription drug costs are typically covered through private drug insurers or publicly managed provincial drug plans. This analysis of drug claims data from Canada was conducted in 2017 by IQVIA Canada (Kirkland, QC, Canada), on behalf of and funded by Pfizer, Inc. (New York City, NY, USA).

Claims data were collated from public and private insurance payers, namely the IQVIA Private Drug Plan (PDP) database, which provides data for approximately 70% of the total private drug claims in Canada, the Ontario Public Drug Plans (OPDP) database and the Quebec Public Drug Plan (Régie de l’assurance maladie du Québec [RAMQ]) database. Ontario and Quebec are home to approximately 61% of the Canadian population. The databases are actively managed and quality-controlled to capture patient demographics, drugs dispensed (including dosage, quantity, and number of days’ supply), service date and place, payer information, and prescribing physician specialty. The OPDP database contains data on all 3.2 million active claimants enrolled in this public drug plan, while the RAMQ drug plan covers approximately 2 million active claimants residing in Quebec. Per privacy legislation in Quebec, a random sample drawn from the total RAMQ database was used in this study. These databases have been utilized previously in many studies evaluating the use of biologics and other medications in adult and pediatric patient populations [7, 8, 25–29].

### Data analysis

The study utilized longitudinal data spanning January 2007 to April 2017. Patients < 18 years of age were indexed when filling a prescription for ETN between January 2008 and January 2016 (index date). Those with biologic treatment in the previous 12 months, aged 17 years (and who would therefore reach 18 years of age during the study), with a drug history indicative of Crohn’s disease, irritable bowel disease, or psoriasis, those whose location was unknown, who were new to the plan at index date, or who were not active in the plan at 15 months following index date were excluded.

**Fig. 1:**
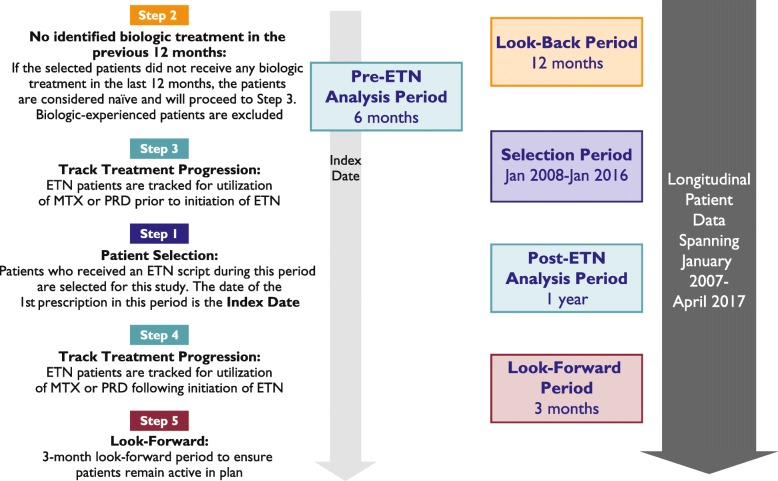
Patient Selection Procedure and Timelines. ETN, etanercept; MTX, methotrexate; PRD, prednisone

Treatment with MTX and PRD was tracked during the 6 months prior to and 12 months following the index date. A schematic of the patient selection procedure is presented in Fig. 1.

Data were categorized by province and/or region, as follows: British Columbia, Alberta, the Prairies (consisting of Manitoba and Saskatchewan), Ontario, Quebec, and the East (consisting of New Brunswick, Nova Scotia, Prince Edward Island, and Newfoundland, and Labrador). The disease indications reported in Table 1 were inferred from a patient’s drug prescription history using an algorithm developed by IQVIA, which utilizes information on medication claims and prescriber types in a patient's history. [8, 30]. The algorithm was developed using a review of Canadian treatment guidelines, comparison to diagnosis-containing datasets, and input from treating experts to identify prescription and prescriber patterns that align with JIA and aims to exclude other diseases that ETN is indicated for, including psoriasis, rheumatoid arthritis, psoriatic arthritis, and ankylosing spondylitis. The algorithm does not identify different JIA categories. This algorithm and approach have been validated [30] and used in other published studies [8].

**Table 1:** Patient characteristics at index date, *n* (%)

Characteristic	Patients (N = 330)
Sex	
Female	222 (67%)
Male	108 (33%)
Age at index	
1–4 years	42 (13%)
5–9 years	77 (23%)
10–14 years	129 (39%)
15–17 years	82 (25%)
Indication*	
Juvenile idiopathic arthritis	316 (96%)
Psoriatic arthritis	10 (3%)
Ankylosing spondylitis	3^†^ (1%)
Region	
Quebec	118 (36%)
Ontario	110 (33%)
Alberta	50 (15%)
East	33 (10%)
British Columbia	15 (5%)
Prairies	3* (1%)
Payer	
PDP	286 (87%)
OPDP	28 (8%)
RAMQ	16 (5%)

* Indications were inferred using an algorithm developed by IQVIA, which uses a patient’s drug history [7, 8, 30]

^†^Groups with less than 6 patients/claims were adjusted to 3 to maintain privacy

PDP, IQVIA Private Drug Plan; OPDP, Ontario Public Drug Plans; RAMQ, Quebec Public Drug Plan, Régie de l’assurance maladie du Québec

Since the RAMQ database reports claims by the age categories of 1–4, 5–9, and 10–14 years, rather than the age of the patient in each claim, data from the other two databases were summarized in these age groups as well. In accordance with IQVIA Canada privacy and confidentiality policies, any data group that contained < 6 patients was blinded to 3. Weekly ETN dose was estimated for patients who completed 12-month continuous ETN therapy (7 x [mg dispensed/days between claims]). Categorical variables were recorded as frequency and percentages. A paired *t*-test was used to compare the drug doses of MTX or PRD before and after index date. *P*-values of < 0.05 were considered significant. All statistical analyses were undertaken using SAS version 9.2 (SAS Institute Inc., Cary, North Carolina, USA).

## Results

### Patients

**Fig. 2:**
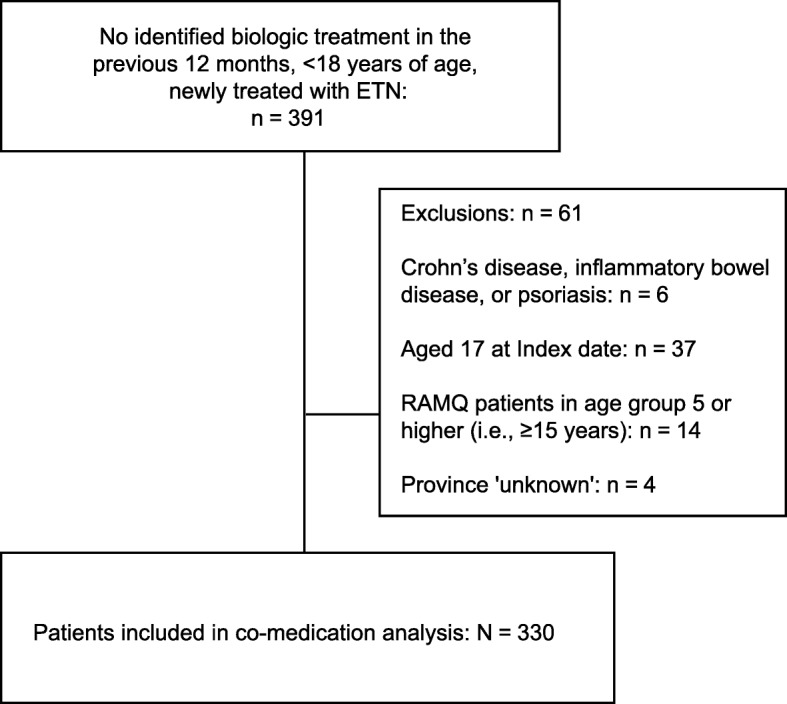
Patient Selection. ETN, etanercept; RAMQ, Quebec Public Drug Plan

Longitudinal claims data for 330 patients who had not received treatment with a biologic in the previous 12 months and who were initiating ETN therapy were included in the analysis (Table 1). Data for a further 61 patients who had not received treatment with a biologic in the previous 12 months and who were patients initiating ETN were identified in the databases but met the pre-specified exclusion criteria (Fig. 2). Most of the patients were excluded for being older than the pediatric age requirement (age 17 or less at index) for this study (Fig. 2).

The demographic and clinical characteristics of the entire population (*n* = 391) before patients were excluded was similar to the group included e.g., 65% female, 94% with a drug history of JIA and 37 and 32% residing in Quebec or Ontario, respectively. The majority of patients included in the study (*n* = 330) were female (67%), aged 10–17 years (64%), and with a drug history consistent with JIA (96%). Most were from Quebec (36%) or Ontario (33%), and were insured through PDP (87%).

### Use of etanercept

**Fig. 3:**
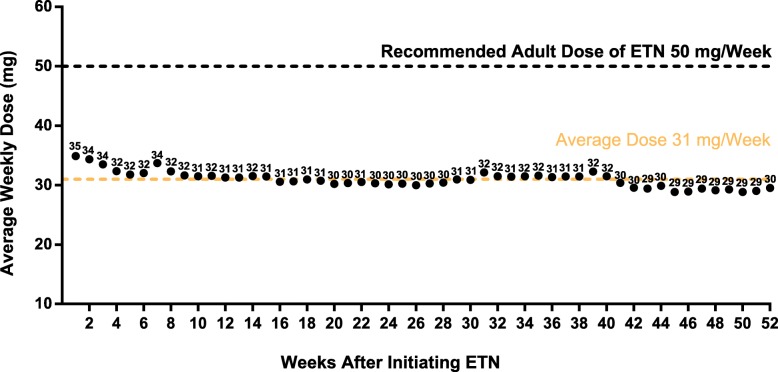
Average Weekly Doses of Etanercept Over the First Year of Therapy for All Indications* ETN, etanercept. *All indications refers to patients with a drug history representing juvenile idiopathic arthritis, psoriatic arthritis, or ankylosing spondylitis. Indications were inferred using an algorithm developed by IQVIA, which utilize a patient’s drug history [7, 8, 30]

**Fig. 4:**
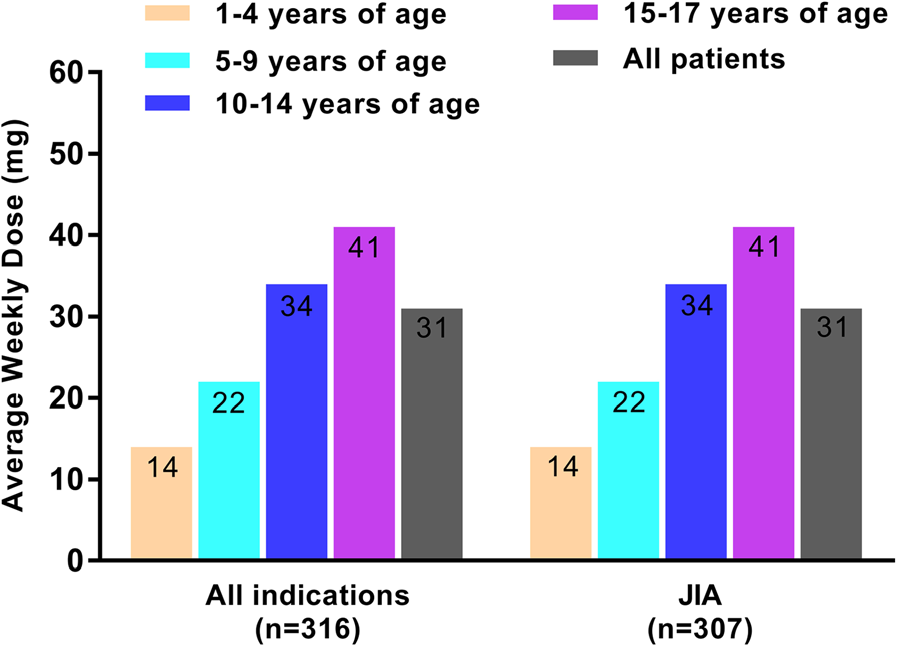
Average Weekly Doses of Etanercept by Age and Indication*. JIA, juvenile idiopathic arthritis. *Indications were inferred using an algorithm developed by IQVIA, which utilizes a patient’s drug history [7, 8, 30]. For psoriatic arthritis and ankylosing spondylitis indications, group sizes were less than 6 per age group. All indications refers to patients with a drug history representing juvenile idiopathic arthritis, psoriatic arthritis, or ankylosing spondylitis

The mean weekly initiation dose of ETN among the 316 patients who completed 12 months of continuous ETN therapy was 35 mg. Over the first year of treatment, the mean dose remained relatively consistent (31 mg per week; Fig. 3). As expected from the weight-based dosing in pediatric patients, the weekly dose of ETN was higher in older patients, ranging from 14 mg in patients 1–4 years of age to 41 mg in those 15–17 years of age (Fig. 4).

### Use of methotrexate and prednisone alongside etanercept

Of the 330 patients who initiated ETN, 222 (67%) did not use MTX, and 223 (68%) did not use PRD, before or after starting ETN (Table 2).

**Table 2:** Co-medication with methotrexate or prednisone with etanercept

Methotrexate Co-Therapy Study	Patients (N = 330)
Received no co-medication before or in the 12 months after ETN initiation	222 (67%)
Received co-medication with MTX before ETN initiation	103 (31%)
Stopped MTX when ETN was initiated	18
Continued MTX for 12 months after ETN initiation	85
Started MTX co-medication after ETN was initiated	3* (2%)
Prednisone Co-Therapy Study	Patients (N = 330)
Received no co-medication before or in the 12 months after ETN initiation	223 (68%)
Received co-medication with PRD before ETN initiation	92 (28%)
Stopped PRD when ETN was initiated	46
Continued PRD for 12 months after ETN initiation	46
Started PRN co-medication after ETN was initiated	15 (5%)

*Groups with less than 6 patients/claims were adjusted to 3 to maintain privacy

ETN, etanercept; MTX, methotrexate; PRD, prednisone

**Fig. 5:**
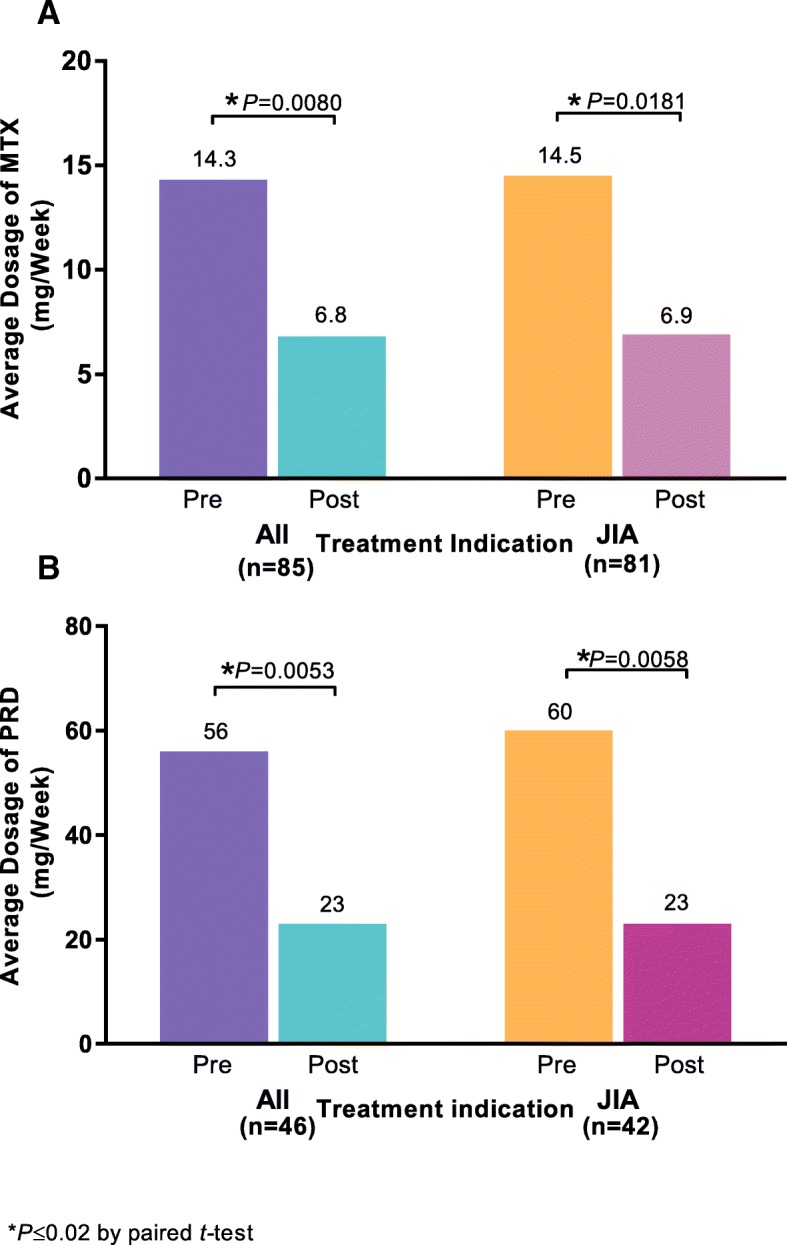
Mean Dose of (**a**) Methotrexate or (**b**) Prednisone Pre- and Post-initiation of Etanercept*. JIA, juvenile idiopathic arthritis; MTX, methotrexate; PRD, prednisone. Indications were inferred using an algorithm developed by IQVIA, which utilizes a patient’s drug history [7, 8, 30]. *These results are from the patients who continued taking these co-medications after the initiation of etanercept. All indications refers to patients with a drug history representing juvenile idiopathic arthritis, psoriatic arthritis, or ankylosing spondylitis

A total of 17% (18/103) of MTX-treated and 50% (46/92) of PRD-treated patients discontinued use of those medications upon ETN initiation (Table 2). The rest continued receiving MTX or PRD within the first 12 months of ETN treatment. Those patients who continued taking MTX or PRD significantly decreased usage of those medications following initiation of ETN (MTX: from 14.3 mg/week to 6.8 mg/week; PRD: 56 mg/week to 23 mg/week), across all indications (Fig. 5). Few patients initiated MTX or PRD therapy during the first year of taking ETN (Table 2).

## Discussion

In this study of Canadian claims-level data, many patients stopped MTX or PRD treatment when ETN was initiated, and those who continued treatment showed a significant reduction in dose. To the best of our knowledge, this is the first large study, specifically designed to evaluate the potential steroid- and MTX-sparing effect of ETN in pediatric patients in real-world practice. This finding is in agreement with previous studies, mostly conducted in adults with rheumatoid arthritis, which showed a steroid-sparing effect of TNF inhibitor therapy [9–14, 31]. Our study therefore helps strengthen and extend the knowledge base in this area. Furthermore, the reduction in steroid requirement can occur quickly. For example, a French study in adults with rheumatoid arthritis demonstrated that 76% of patients reduced their PRD use during the first 3 months of anti-TNF therapy, and 15% discontinued PRD use altogether [32]. Similarly, a study conducted in the Netherlands, which enrolled 94 patients with recent onset non-systematic JIA demonstrated that among the patients treated with a combination of ETN and MTX over the first 3 months of treatment no patients required additional treatment with corticosteroids, whereas 10% of patients receiving initial treatment with MTX or sulfasazine required the use of corticosteroids [33]. Tzaribachev et al described the results of a small (*n* = 25) German registry study, which identified children below four years’ of age who had initiated treatment with ETN. In this German study, 24 patients (96%) were being treated with MTX and 21 (84%) with PRD or methyl-PRD when they initiated treatment with ETN [34]. After a mean of 19 months of ETN treatment, five patients in the German had stopped taking MTX (21% of those receiving MTX at baseline) and 3 patients had discontinued treatment with PRD (14% of those receiving PRD at baseline) [34]. The proportion of patients discontinuing MTX in the German study was similar to that in our study (21% vs 17%, respectively) whereas a much smaller proportion discontinued PRD (14% vs 50% in our study). This difference might be related to the differences in the age of the patients being included in this study. Just 13% of patients in our study were aged 1–4 years, whereas all of the patients in the German registry study were aged less than 4 years. In addition, and similar to our study, a small Dutch cost-effectiveness study of children with JIA (*n* = 49) found a reduction in the proportion of patients using both glucocorticoids (61 to 47%) and MTX (100 to 80%) following the initiation of ETN [35]. A recent study conducted in Russia, which followed 215 patients with JIA treated with either MTX, ETN, or MTX and ETN combination therapy, demonstrated that over 80% of patients receiving combination therapy, and glucocorticoids at the start of the study experienced a reduction in the dose or cessation of glucocorticoids. [36]. Most patients treated with ETN, either alone or in combination with MTX, were also able to discontinue treatment with NSAIDs: more than 85% of patients stopped NSAIDS within 3 months of starting ETN + MTX [36].

A large pharmacovigilance study evaluating data from 15,284 patients with JIA from three registries (Pharmachild which is international, BiKeR for Germany and Austria, and a Swedish registry) found that MTX was the most commonly used first-choice synthetic DMARD, used by 61% of patients in Sweden and 84% of patients in the BiKer and Pharmachild registries [37]. Among the biologics, ETN was the most frequently used across the three registries, with 44% of patients in Pharmachild, 62% in BiKeR, and 24% in the Swedish registry. This widespread use of MTX and ETN in the treatment of JIA suggests that the findings of our study are likely to be relevant to many clinicians managing patients with JIA.

Any reduction in the dosage of MTX or PRD in children is likely to result in fewer side effects, in the case of MTX: nausea, vomiting, elevated liver enzymes, and rarer but more serious effects on bone marrow leading to hematological abnormalities such as leukopenia/neutropenia [38, 39]. A study designed to evaluate persistence with MTX in 577 patients with JIA (median age 9 years at the start of treatment) demonstrated that after 2 years, 54% of patients had discontinued MTX as their sole DMARD, with 25% of these discontinuations due to adverse events [40]. Nausea, vomiting, and elevated liver enzymes were the most common adverse events [40]. For steroids, a reduction in dose may help prevent an increased risk of growth restriction, weight gain, bone demineralization leading to fractures, infection at higher doses and delays in development [20–22, 41, 42]. A cost effectiveness analysis of first-line ETN therapy utilizing data from a systematic review into the use of biologics for the treatment of JIA, [43] suggested that, in patients with JIA, the rate of adverse events is double with MTX versus ETN therapy [44]. The side effects of steroids are often associated with an increased requirement for medical supervision and treatments, such as the use of growth hormone or treatment of fractures [21, 22].

Pediatric patients with JIA who have not responded to conventional DMARDs such as MTX, and who subsequently benefit from treatment with TNF inhibitors, are likely to have improvements in quality of life [35, 43–45]. Although TNF inhibitors are more expensive than steroids or synthetic DMARDs, clear benefits are gained in quality of life, increases in productivity, and reductions in healthcare utilization, at least in the adult population [46–48]. Formal cost-effectiveness assessments of TNF inhibitors in pediatric JIA patients have shown that switching inadequate responders or non-responders from MTX treatment is associated with better short-term outcomes but at a relatively high cost per quality-adjusted life year (QALY) [35, 43–45]. A Markov modelling analysis of the cost effectiveness of first-line biologic therapy with ETN for JIA based on the Canadian healthcare system suggested that the incremental costs of first-line ETN versus usual care was $16,893 per QALY gained [44]. However, with the cost of a severe adverse event of $7817, this margin would be reduced approximately 2.5-fold [44]. Furthermore, active disease was judged to be over twice as expensive as quiescent disease ($3930 vs $1702), with the likelihood of quiescence being approximately twice that in patients receiving MTX and ETN versus those receiving MTX alone [44]. The healthcare costs associated with ETN, therefore, are clearly higher, but may be necessary due to high disease activity in patients who fail to respond to MTX.

Some of the pediatric patients included in our study were approaching adulthood. The transfer between pediatric and adult care for patients with rheumatic disease can be complex and has been shown to lead to an increase in active disease in 30% of patients [49–51]. The importance of uninterrupted care has led the American College of Rheumatology to develop a tool kit for healthcare professionals aimed at facilitating transition of patients through this potentially difficult period [52].

Although the costs associated with poor adherence with or switching medications in pediatric patients with JIA is not well studied, in adult patients with RA, switching or non-adherence are associated with higher overall healthcare costs [53, 54]. A recent study of long-term treatment retention in Canadian adults initiating therapy with ETN reported that yearly retention rates are high, particularly after the first year of treatment (66% in year 1 and 79–84% in years 2–6) [8]. In a similar study in the same setting, yearly retention rates for pediatric patients (2 to 16 years) initiating ETN were even higher than those previously reported for adults (78% in year 1 and 80–90% over years 2–6) [7]. In this pediatric retention study, we did not see any significant evidence or effects of drug switching as pediatric patients approached adulthood.

The limitations of this study are inherent to its design. In common with all retrospective cohort studies utilizing claims data, the following limitations apply: restricted information being available on disease activity and severity, comorbid conditions and other clinical or socioeconomic characteristics. Therefore, caution should be used when interpreting these findings because adjusting for all bias and potential confounding factors is not possible. Additional limitations included that the indication for which ETN was prescribed was inferred from prior prescription data, and may not match the actual JIA category. This also means that we cannot look at any differences between JIA categories. We were also unable to ascertain the exact reasons for changes in co-medication use. Furthermore, the study was not designed to track longitudinal data on MTX or PRD persistence. Instead, it used a binary start/stop approach to signalling the use of co-medication, relative to the index date. Further study would be needed to examine trends over time in cessation of co-medication after ETN initiation. A 12-month look-back period was utilized in this study to examine the use of MTX or PRD before the initiation of ETN. Patients may have received these medications before this look-back period but they would not be included in the received no co-medication grouping. Nevertheless, the strength of this study is that it uses real-world data reflecting actual clinical practice from a large geographically diverse patient population. Furthermore, the algorithm we used has been utilized in a number of studies previously [7, 8, 30].

## Conclusions

This study of Canadian claims-level data demonstrates that the introduction of ETN therapy corresponds with a decline in MTX and PRD use and dosage in pediatric patients with rheumatic diseases, predominantly those with a medication history consistent with JIA. Reductions in the use of concomitant medications may be beneficial in terms of safety, tolerability, and overall healthcare costs.

## Data Availability

Upon request, and subject to certain criteria, conditions and exceptions (see https://www.pfizer.com/science/clinical-trials/trial-data-and-results for more information), Pfizer will provide access to individual de-identified participant data from Pfizer-sponsored global interventional clinical studies conducted for medicines, vaccines and medical devices [1] for indications that have been approved in the US and/or EU or [2] in programs that have been terminated (i.e., development for all indications has been discontinued). Pfizer will also consider requests for the protocol, data dictionary, and statistical analysis plan. Data may be requested from Pfizer trials 24 months after study completion. The de-identified participant data will be made available to researchers whose proposals meet the research criteria and other conditions, and for which an exception does not apply, via a secure portal. To gain access, data requestors must enter into a data access agreement with Pfizer.

## Abbreviations

DMARD: Disease-modifying anti-rheumatic drugs
ETN: Etanercept
JIA: Juvenile idiopathic arthritis
MTX: Methotrexate
OPDP: Ontario Public Drug Plans
PDP: IQVIA Private Drug Plan
PRD: Prednisone
QALY: Quality-adjusted life year
RA: Rheumatoid arthritis
RAMQ: Quebec Public Drug Plan, Régie de l’assurance maladie du Québec
TNF: Tumor necrosis factor
